# A very rare phenotype of immunoglobulin G4-related disease that was manifested as constrictive pericarditis: a case report

**DOI:** 10.1093/ehjcr/ytae689

**Published:** 2024-12-30

**Authors:** Kenshi Ono, Tetsuya Nomura, Keisuke Shoji, Yukinori Kato, Naotoshi Wada

**Affiliations:** Department of Cardiovascular Medicine, Kyoto Chubu Medical Center, 25, Yagi-Ueno, Yagi-cho, Nantan City, Kyoto 629-0197, Japan; Department of Cardiovascular Medicine, Kyoto Chubu Medical Center, 25, Yagi-Ueno, Yagi-cho, Nantan City, Kyoto 629-0197, Japan; Department of Cardiovascular Medicine, Kyoto Chubu Medical Center, 25, Yagi-Ueno, Yagi-cho, Nantan City, Kyoto 629-0197, Japan; Department of Cardiovascular Medicine, Kyoto Chubu Medical Center, 25, Yagi-Ueno, Yagi-cho, Nantan City, Kyoto 629-0197, Japan; Department of Cardiovascular Medicine, Kyoto Chubu Medical Center, 25, Yagi-Ueno, Yagi-cho, Nantan City, Kyoto 629-0197, Japan

**Keywords:** Case report, IgG4-related disease, Constrictive pericarditis, Pericardiectomy, Echocardiogram

## Abstract

**Background:**

Constrictive pericarditis (CP) can arise from various causes, including post-operative degeneration, tuberculosis, and sequelae of pericarditis. Immunoglobulin (Ig) G4-related disease is a rare but recognized cause of CP. However, the specific mechanisms underlying these aetiologies and pathologies remain unclear.

**Case summary:**

A 67-year-old man presented with a 6-month history of bilateral leg oedema, anorexia, and dyspnoea on exertion. Computed tomography (CT) revealed significant pericardial thickening without calcification, right pleural effusion, and ascites. Echocardiography demonstrated a reduced left ventricular ejection fraction and pericardial thickening. The early diastolic mitral annular tissue velocity (e′) was preserved as 11.7 cm/s, despite inferior vena cava dilation. Respiratory variations in mitral inflow velocities and septal bounces were unremarkable. Cardiac catheterization further showed a ‘dip and plateau’ pattern with equalization of bilateral ventricular end-diastolic pressure. A preliminary diagnosis of CP was made, and pericardiectomy was performed, increasing the cardiac index from 2.0 to 3.0 L/min/m^2^. Pathological examination revealed marked IgG4-positive plasma cell infiltration and tissue fibrosis. Additionally, the patient’s post-operative serum IgG4 level was 679 mg/dL. Given these findings, IgG4-related CP without involvement of other organs was determined as the definitive diagnosis. His clinical status improved without requiring corticosteroid therapy.

**Discussion:**

Optimal therapy for IgG4-related CP remains elusive due to its rarity. Potential therapeutic options include pericardiectomy, pericardiotomy, and corticosteroid therapy. Further examination through the accumulation of similar cases is crucial to establish definitive treatment approaches for this condition.

**Learning points**

- IgG4-related disease should be considered as one of the causes of constrictive pericarditis which is a potentially curable cause of heart failure.
- IgG4-related disease has the possibility of affecting wide variety of cardiovascular system and the optimal therapy for IgG4-related constrictive pericarditis remains elusive.

## Introduction

Constrictive pericarditis (CP) is a pathological state characterized by pericardial thickening and inelasticity, leading to impaired diastolic filling and elevated diastolic pressures resulting in right-sided heart failure. Various factors can contribute to CP, including tuberculosis, irradiation, or post-operative complications.^1^ Interestingly, immunoglobulin (Ig) G4-related disease, a systemic condition of serum IgG4 levels, significant IgG4-positive plasma cell infiltration, and tissue fibrosis, has been implicated as a cause of CP.^2^ While the aorta is typically affected in the cardiovascular involvement of IgG4-related disease, a wider range of cardiovascular structures have also been documented to be affected.^3,4^

## Summary figure

**Figure:**
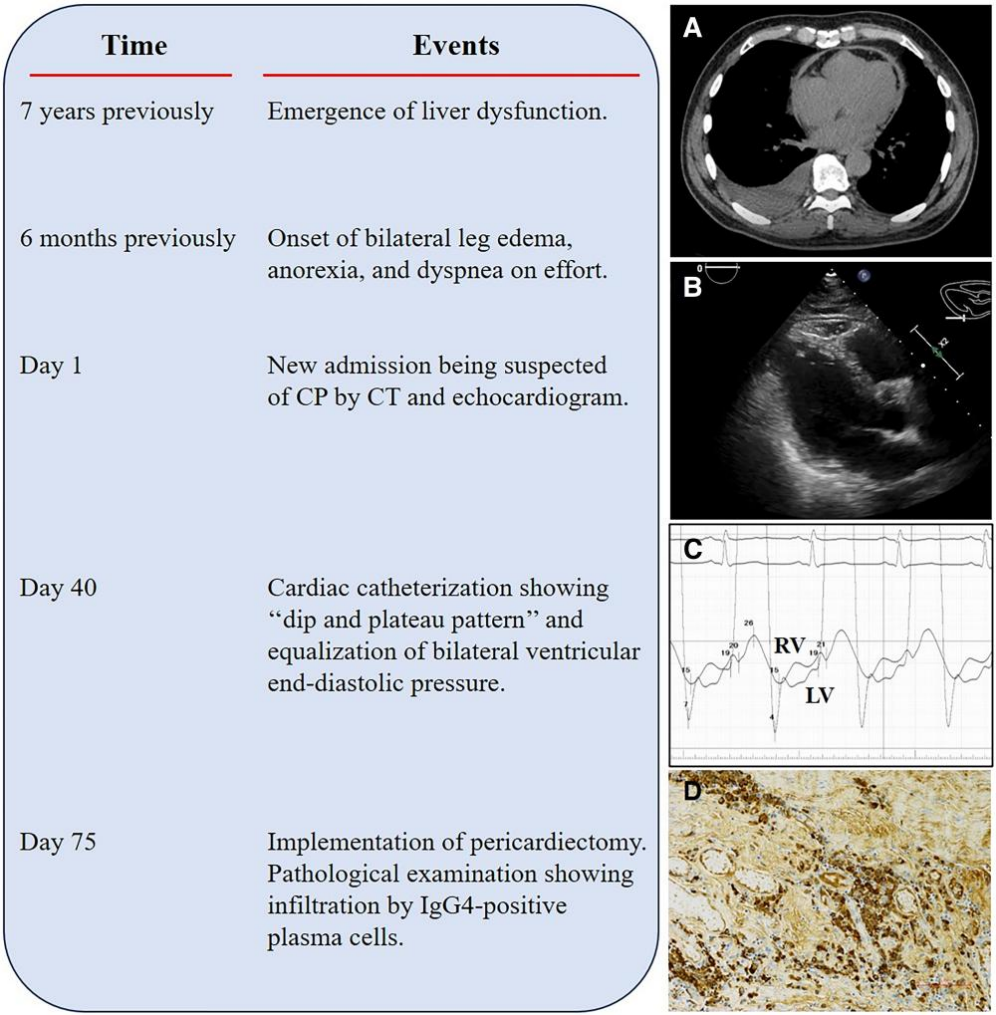
Images on the right display. (*A*) Right pleural effusion and significant pericardial thickening without calcification are seen on computed tomography. (*B*) Reduced right ventricular ejection fraction, diastolic dysfunction, and pericardial thickening are seen on echocardiography. (*C*) A ‘Dip and plateau’ pattern with equalization of bilateral ventricular end-diastolic pressures are revealed on hemodynamic cardiac catheterization. (*D*) Increased counts of IgG4-positive plasma cells are seen on immunostaining of the resected pericardium.

## Case presentation

A 67-year-old male with a 7-year history of liver dysfunction presented with a 6-month history of bilateral leg oedema, anorexia, and dyspnoea on exertion. The patient’s medical history was unremarkable for irradiation, tuberculosis infection, or surgical procedures. On admission, vital signs were normal (blood pressure, 118/70 mmHg; pulse rate, 97 beats per minute), and physical examination demonstrated conjunctival icterus, jugular venous distention, and bilateral pitting leg oedema. Pericardial friction rubs were not auscultated. Routine laboratory examination showed liver dysfunction (alkaline phosphatase, 611 U/L [115–359 U/L]; total-bilirubin, 2.56 mg/dL [0.30–1.20 mg/dL]; gamma-glutamyl transferase, 224 U/L [0–70 U/L]), and mildly elevated brain natriuretic peptide levels (68.3 pg/mL).

**Figure 1:**
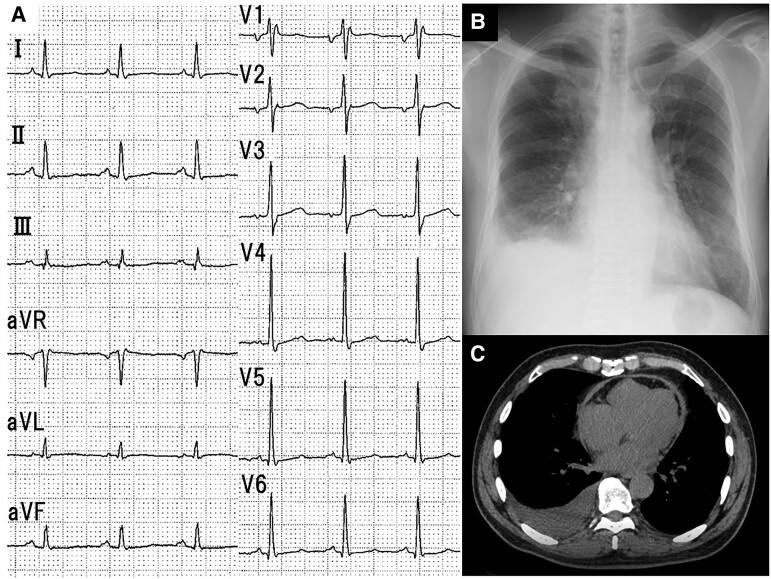
(*A*) Electrocardiography shows sinus rhythm and prominent mitral P waves. (*B*) Mild cardiomegaly and a right blunt costophrenic angle are identified on chest radiography. (*C*) Computed tomography shows right pleural effusion and significant pericardial thickening without calcifications.

**Figure 2:**
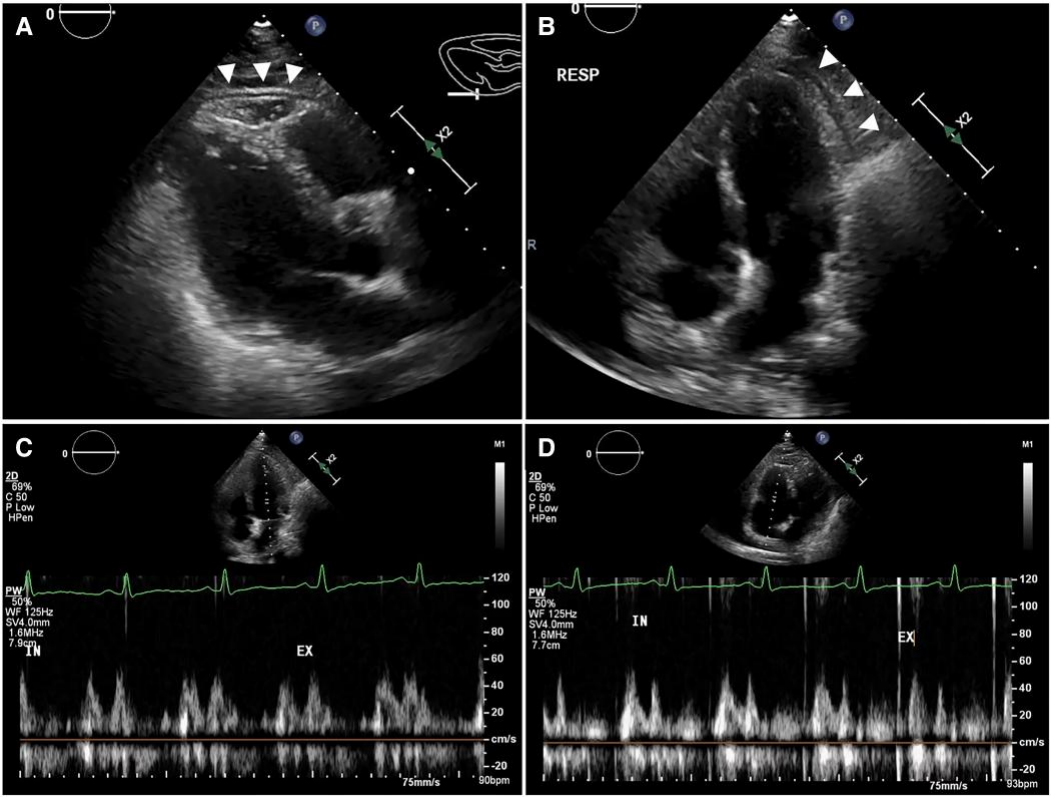
(*A*, *B*) Ultrasound echocardiography shows reduced right ventricular ejection fraction, diastolic dysfunction, and pericardial thickening (arrowheads). Respiratory variations are absent in the transmitral (*C*) and transtricupsid flow patterns (*D*).

Electrocardiography showed sinus rhythm with prominent mitral P waves (*Figure 1A*). Chest radiography revealed a cardiothoracic ratio of 52.7% and a blunted right costophrenic (*Figure 1B*). Computed tomography (CT) identified right-sided pleural effusion, ascites, and significant pericardial thickening without calcification (*Figure 1C*). Furthermore, ultrasound echocardiography showed a reduced left ventricular ejection fraction (47%) and pericardial thickening (*Figure 2A* and *B*, arrowheads; Supplementary material online; Supplementary material online, *Videos S1* and *S2*). The inferior vena cava (IVC) appeared dilated with no respiratory variation. Notably, the septal early diastolic mitral annular tissue velocity (medial e′) was preserved (11.7 cm/s), but was lower than the lateral e′ (12.8 cm/s). Neither remarkable respiratory septal shift (septal bounce) nor significant respiratory variations were observed in the transmitral (*Figure 2C*) and transtricupsid flow patterns (*Figure 2D*). In addition, the four-chamber view demonstrated abnormal beat-to-beat ventricular septal motion. While these echocardiographic findings were not entirely characteristic of CP, his clinical presentation suggested right-sided heart failure.

**Figure 3:**
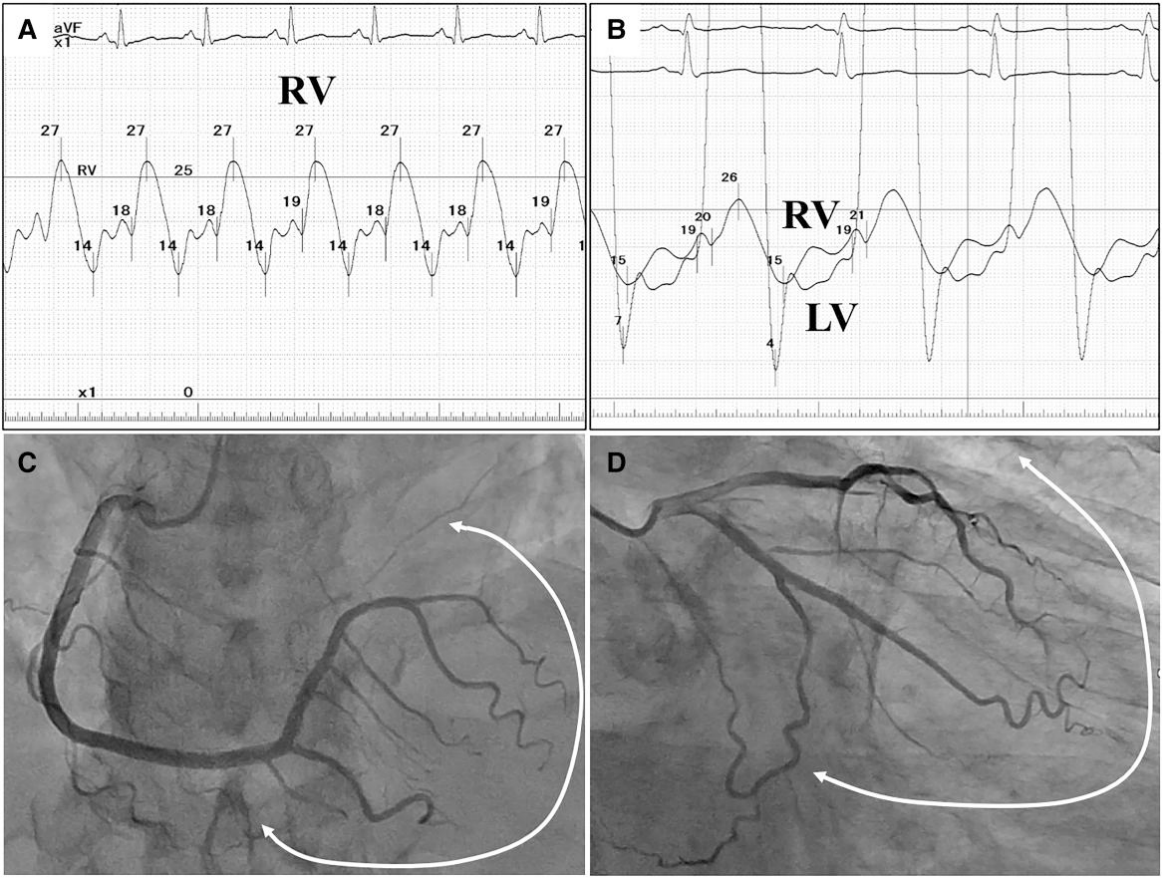
Haemodynamic cardiac catheterization demonstrates a ‘dip and plateau’ pattern (*A*) and equalization of bilateral ventricular end-diastolic pressure (*B*). (*C*, *D*) Coronary angiography shows absent organic stenoses and highly fixed, unsynchronized coronary arteries (arrow range).

Haemodynamic cardiac catheterization revealed a ‘dip and plateau’ pattern (*Figure 3A*) with equalization of the bilateral ventricular end-diastolic pressures (*Figure 3B*). Moreover, elevated right atrial pressure (17 mmHg), slightly increased pulmonary arterial wedge pressure (16 mmHg), and decreased cardiac index (1.6 L/min/m^2^) were identified. These findings were collectively consistent with CP and right-sided heart failure. Coronary angiography identified absent organic stenoses and highly fixed, unsynchronized coronary arteries (*Figure 3C* and *D*, Supplementary material online, *Videos S3* and *S4*). With no evident cause for the pericardial thickening (idiopathic), diuretics were initiated, resulting in modest but insufficient improvement of symptoms.

**Figure 4:**
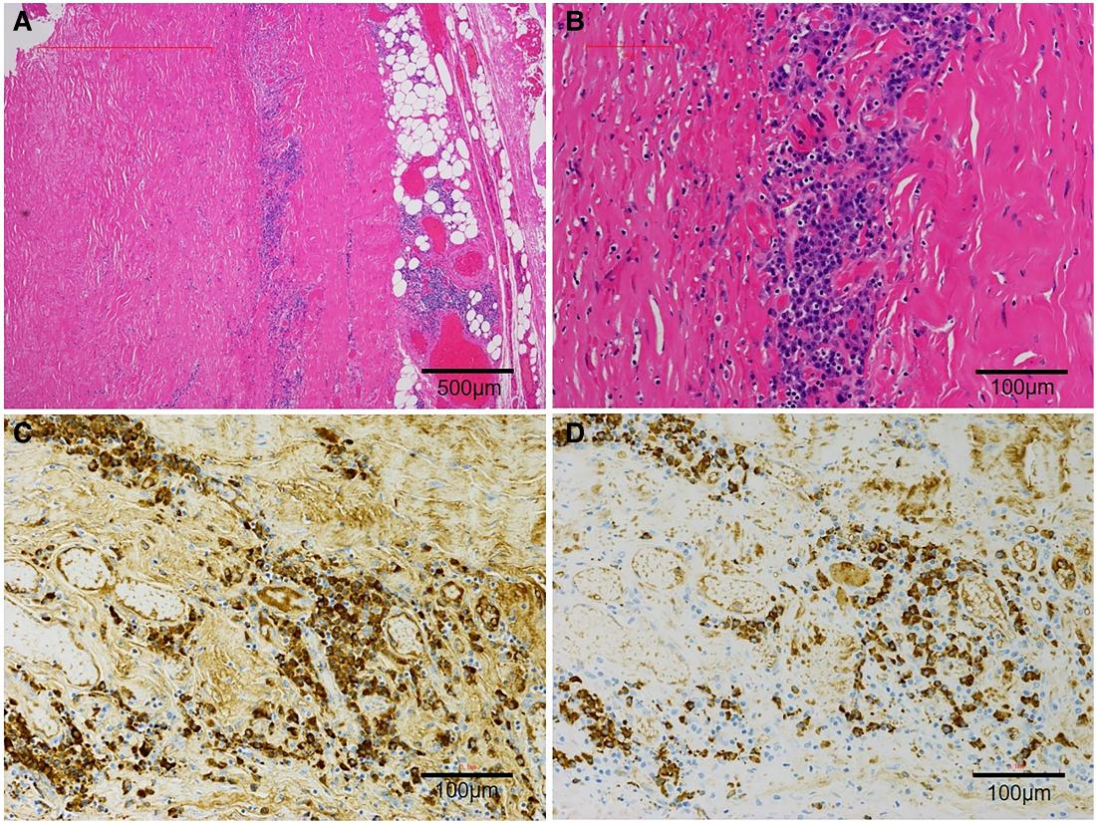
(*A*, *B*) Haematoxylin and eosin staining of the resected pericardium shows partial lymphocyte infiltration and prominent fibrosis. (*C*, *D*) Immunostaining demonstrates an increased count of IgG4-positive plasma cells.

Our heart team discussed about the treatment strategy for this pathological entity. Concludingly, surgical treatment was adopted as the diagnosis of CP with unknown aetiology and refractory to medical therapy. Intraoperative hemodynamic monitoring demonstrated that cardiac index was remarkably increased from 2.0 to 3.0 L/min/m^2^ by conducting pericardiectomy. The patient’s clinical symptoms improved without diuretics, and liver function tests were normalized in 1 week after surgery. Post-operative echocardiography confirmed the resolution of abnormal beat-to-beat ventricular septal motion and IVC dilation. Pathological examination of the resected pericardium revealed partial lymphocyte infiltration and prominent fibrosis (*Figure 4A* and *B*). Immunostaining demonstrated a substantial increase in IgG4-positive plasma cells (70 cells per high-power field in the most affected area) and an elevated IgG4/IgG-positive cell ratio (≤70%) (*Figure 4C* and *D*). Further laboratory testing revealed highly elevated serum IgG4 levels (679 mg/dL). Based on the comprehensive diagnostic criteria for IgG4-related diseases, a definitive diagnosis of IgG4-related CP was made. Given the absence of other organ involvement, corticosteroid therapy was not initiated. The patient’s post-operative course remained uneventful. There has been no recurrence for more than 3 years since the surgery.

## Discussion

This report describes a rare case of IgG4-related CP that was successfully treated with pericardiectomy. IgG4-related disease, first recognized in 2001 as autoimmune pancreatitis with elevated IgG4 levels,^5^ is a distinct fibro-inflammatory disorder driven by immune-mediated processes. In the case of the pericardium, inflammation of the visceral and parietal layers can cause fibrosis, thickening, adhesions, calcifications, and other degenerative changes, ultimately restricting ventricular filling and compromising cardiac function. While uncommon, causes of IgG4-related CP are reported annually. However, the paucity of such cases hinders the establishment of optimal treatment strategies. Therefore, continuous accumulation of similar cases and evaluation of diagnostic processes and treatment outcomes are necessary.^3,6–8^

The clinical presentation of CP is dependent on pericardial constriction severity. Patients typically experience symptoms of right-sided heart failure, including chest discomfort, mild lower extremity oedema, or pleural effusion. As such, early CP diagnosis is often challenging due to the absence of specific symptoms.^9^ Similarly, identifying the underlying cause of CP can be difficult. Most cases are idiopathic (78%–86%), whereas the remaining cases have been attributed to malignancy (5%–7%), tuberculosis (4%), and autoimmune or post-cardiac surgery (1%–7%).^10^ Rare cases of cardiac storage disease have also been reported to cause CP.^11^

Given the rising prevalence of heart failure worldwide, CP presents as a potentially curable aetiology. Early and accurate diagnosis is therefore essential for patient comfort and prognosis.^12^ Multi-modal imaging, encompassing echocardiography,^13^ cardiac CT, and cardiac magnetic resonance imaging (MRI),^3^ has been shown to be useful in the evaluation and management of CP.^14^ Despite this, several echocardiographic findings in our case deviated from its typical presentation. Nevertheless, haemodynamic cardiac catheterization and physical examination findings supported our diagnosis. The imaging evidence of pericardial inflammation by cardiac MRI might be helpful to identify potentially reversible forms of constrictive pericardium for which empiric anti-inflammatory therapy was considered and might have prevented the need for pericardiectomy. In that sense, we should have evaluated cardiac MRI. However, we also considered essential to elucidate the accurate aetiology in our case rather than simply conducting empiric therapy.

Pericardiectomy is generally considered the first-line treatment for CP. Pericardial window surgery, a less invasive approach, has also emerged as a potential treatment strategy.^15^ Approximately, half of IgG4-related pericarditis cases progress to CP, for which steroids, pericardial window surgery, or a combination of both have been explored as treatment options. Early diagnosis of IgG4-related CP in our case might have prompted initial corticosteroid therapy, resolving the clinical condition without requiring surgery.

In conclusion, IgG4-related disease may affect a diverse range of cardiovascular structures. While several therapeutic options have been explored by previous studies and in this case report, the optimal treatment approach for such cases remains unclear. Further investigation through the accumulation of similar cases is necessary to establish definitive treatment strategies.

## Supplementary Material

ytae689_Supplementary_Data

## Data Availability

The data underlying this article are available in the article and in its online Supplementary material.

## Lead author biography

Dr Kenshi Ono is a clinical fellow in the Department of Cardiovascular Medicine at Kyoto Chubu Medical Center. He specializes in interventional cardiology and endovascular treatment.

## Supplementary material

**Consent:** The authors confirm that written consent for submission and publication of this case report including images and associated text has been obtained from the patient in line with COPE guidance.

**Funding:** None declared.
